# Oligomeric amyloid beta prevents myelination in a clusterin-dependent manner

**DOI:** 10.21203/rs.3.rs-4415143/v1

**Published:** 2024-05-30

**Authors:** Rebecca M. Beiter, Tula P. Raghavan, Olivia Suchocki, Hannah E. Ennerfelt, Courtney R. Rivet-Noor, Andrea R. Merchak, Jennifer L. Phillips, Tim Bathe, John R. Lukens, Stefan Prokop, Jeffrey L. Dupree, Alban Gaultier

**Affiliations:** UMass Chan Medical School; University of Virginia School of Medicine; University of Virginia School of Medicine; University of Virginia School of Medicine; University of Virginia School of Medicine; University of Virginia School of Medicine; University of Florida; University of Florida; University of Virginia School of Medicine; University of Florida; Virginia Commonwealth University; University of Virginia School of Medicine

**Keywords:** Oligodendrocyte progenitor cells, clusterin, myelin, Alzheimer’s Disease, IL-9

## Abstract

**Background:**

White matter loss is a well-documented phenomenon in Alzheimer’s disease (AD) patients that has been recognized for decades. However, the underlying reasons for the failure of oligodendrocyte progenitor cells (OPCs) to repair myelin deficits in these patients remain elusive. A single nucleotide polymorphism (SNP) in Clusterin has been identified as a risk factor for late-onset Alzheimer’s disease and linked to a decrease in white matter integrity in healthy adults, but its specific role in oligodendrocyte function and myelin maintenance in Alzheimer’s disease pathology remains unclear.

**Methods:**

To investigate the impact of Clusterin on OPCs in the context of Alzheimer’s disease, we employed a combination of immunofluorescence and transmission electron microscopy techniques, primary culture of OPCs, and an animal model of Alzheimer’s disease.

**Results:**

Our findings demonstrate that Clusterin, a risk factor for late-onset AD, is produced by OPCs and inhibits their differentiation into oligodendrocytes. Specifically, we observed upregulation of Clusterin in OPCs in the 5xFAD mouse model of AD. We also found that the phagocytosis of debris, including amyloid beta (Aβ), myelin, and apoptotic cells leads to the upregulation of Clusterin in OPCs. In vivo experiments confirmed that Aβ oligomers stimulate Clusterin upregulation and that OPCs are capable of phagocytosing Aβ. Furthermore, we discovered that Clusterin significantly inhibits OPC differentiation and hinders the production of myelin proteins. Finally, we demonstrate that Clusterin inhibits OPC differentiation by reducing the production of IL-9 by OPCs.

**Conclusion:**

Our data suggest that Clusterin may play a key role in the impaired myelin repair observed in AD and could serve as a promising therapeutic target for addressing AD-associated cognitive decline.

## Background

Alzheimer’s Disease (AD) is a neurodegenerative disease with limited therapeutic options to effectively prevent disease progression, and patients inevitably succumb to debilitating dementia [1]. White matter loss has been documented in AD patients, but how this myelin loss contributes to disease progression remains unclear [2]. Recent studies indicate that generation of new myelin-forming oligodendrocytes is critical for memory consolidation and recall [3, 4]. Additionally, remyelination therapeutics have been shown to reduce cognitive deficits seen in an animal model of AD [5]. This evidence supports the hypothesis that the inability to generate oligodendrocytes and maintain myelin is a significant driver of the cognitive deficits observed in AD.

The adult brain contains oligodendrocyte progenitor cells (OPCs), a highly proliferative population of glia that are maintained in a progenitor state throughout adulthood and are normally capable of generating nascent oligodendrocytes [6, 7]. However, it is not understood why OPCs fail to repair myelin damage present in AD. The ability of OPCs to produce mature, myelinating oligodendrocytes during adulthood is critical, as motor learning, memory consolidation, and memory recall are all dependent on de novo production of myelin [3, 4, 8].

Here we investigated if clusterin, a risk factor for late-onset AD, alters the function of OPCs in AD [9]. Clusterin is a multifunctional apolipoprotein that is upregulated in multiple neurodegenerative disorders and is known to play a role in the clearance of debris [10–13]. We have previously reported that in the adult mouse brain a subset of OPCs express Clusterin using single cell transcriptomics [14]. Here, we found that a OPCs in both normal aging and Alzheimer’s patients express clusterin, mimicking single-cell sequencing data from humans [15]. This same data indicates that OPCs, but not any other brain-resident cell type, upregulates clusterin expression in the context of AD [15]. Consequently, we investigated what factors might contribute to clusterin production in OPCs. We found that phagocytosis of debris, including both oligomeric Aβ and myelin debris, results in an upregulation of clusterin production by OPCs. We further discovered that clusterin is a potent inhibitor of OPC differentiation and the production of myelin proteins in vitro. At a mechanistic level, we found that clusterin reduces the production of the cytokine IL-9 by OPCs and that restoration of IL-9 levels rescues the ability of primary OPC culture to differentiate in the presence of clusterin. Deletion of clusterin *in vivo* improved myelination in control and 5XFAD animals, supporting our results showing that clusterin expression is detrimental to the myelination process.

## Material and Methods

### Animals

C57BL/6J (Jackson, #000664) were purchased from Jackson or bred at the University of Virginia. 5xFAD mice (Jackson #34848) were bred at the University of Virginia [16]. Clusterin knockout mice (Jackson #005642) were bred at the University of Virginia. Mice were maintained on a 12-hour light/dark cycle with lights on at 7am. All animal experiments were approved and complied with regulations of the Institutional Animal Care and Use Committee at the University of Virginia (protocol #3918).

### Human Tissue Samples

Human post-mortem brain samples were provided by Dr. Stefan Prokop at the University of Florida’s Neuromedicine Human Brain and Tissue bank. All tissue collection and preparation were approved by the Institutional Review Board at the University of Florida. Clinical details of all human specimens analyzed are details in **Supplemental Table 1**.

### OPC Culture

OPCs were cultured as previously described, with a few modifications [17]. Briefly, postnatal cortices (P0-P4) were rapidly dissected, and meninges removed. The tissue was digested in 2ml of Accutase (Gibco, A1110501) supplemented with 50 units/mL DNase (Worthington Biochemical, LS002139). Cells were then passed through a 70μm filter and grown in suspension in neurosphere media consisting of DMEM/F12 (Gibco, 11320082), B27 (Gibco, 17504044), Pen-Strep (Gibco, 15140122), and 10ng/mL EGF (Peprotech, 315–09). Following expansion as neurospheres, cells were switched to oligosphere media consisting of DMEM/F12 (Gibco, 11320082), B27 (Gibco, 17504044), Pen-Strep (Gibco, 15140122), 10ng/mL FGF (Peprotech, 450 – 33), and 10ng/mL PDGF-AA (Peprotech, 315–17). Cells were allowed to grow in suspension for at least 2 days. Cells were then plated as attached OPCs on 0.01% Poly-L-Lysine coated plates (Electron Microscopy Sciences, 19320-B) in the same media. Cells were allowed to attach for at least 12 hours and then subsequent assays were performed.

### OPC Proliferation

OPCs were plated in proliferation media (described above) supplemented with 8μg/mL clusterin (Sino Biological 50485-M08H) or an equivalent volume of vehicle (H_2_0 or PBS) for the control samples. OPCs were allowed to proliferate for 40–72 hours. Cell number was assessed using the Cell Counting Kit-8 (Dojindo, CK04) according to manufacturer’s instructions. Optical density (OD) was measured at 450nm. For data analysis, the OD of a media only control was subtracted from the OD of all experimental samples. The OD of wells treated with clusterin were then normalized to the biologically identical control well.

### OPC Differentiation

OPCs were plated in proliferation media (described above) or differentiation media consisting of DMEM/F12 (Gibco, 11320082), B27 (Gibco, 17504044), Pen-Strep (Gibco, 15140122), 10ng/mL FGF (Peprotech, 450 – 33), 10ng/mL CNTF (Peprotech, 450 – 13), and 40ng/mL T3 (Sigma, T6397). For clusterin conditions, 8μg/mL clusterin (Sino Biological 50485-M08H) was added to the differentiation media and an equivalent volume of vehicle (H_2_0 or PBS) was added to the differentiation and proliferation control samples. For IL-9 rescue experiments, 100ng/ml IL-9 (Peprotech 219–19) was added to the differentiation media and an equivalent volume of vehicle (0.1% BSA) was added to the differentiation and proliferation control samples. For VEGF inhibition experiments, 10μg/ml of a VEGF function-blocking antibody (R&D Systems MAB9947-SP) was added to differentiation media and 10μg/ml of control IgG (Biolegend 403501) was added to the differentiation and proliferation control samples. OPCs were allowed to differentiate for 48–72 hours and were then subsequently processed for RNA extraction and qPCR or for immunofluorescence.

#### Aβ Preparation and in vitro treatment

Aβ oligomers were prepared as previously described [18]. Briefly, human Amyloid Beta_1–42_ (Echelon Biosciences, 641 – 15) was dissolved in HFIP (Sigma, 52517) to make a 1mM solution and was allowed to desiccate overnight. The resulting peptide film was diluted to a 5mM solution in DMSO and subsequently diluted to a 100μM solution in phenol-free F-12 cell culture media (Gibco, 11039–021) and allowed to incubate overnight at 4°C. For the analysis of clusterin expression following Aβ-treatment, OPCs were treated with 3μM Aβ or a vehicle control for 4 hours. For experiments that included samples treated with CytoD, cells were pretreated for 30 minutes with 1μM CytoD (Millipore-Sigma, C8273) or DMSO and subsequently treated with 3μM Aβ or vehicle control (also containing CytoD or DMSO) for 4 hours. For CypHer-labeled Aβ experiments, 400μM Aβ oligomers in DMSO and phenol-free F-12 cell culture media was incubated with an equivalent volume of 0.1M sodium bicarbonate (Fisher Scientific, S233–500) and 400μM CypHer5e (Cytiva, PA15401) for 30 minutes at room temperature. Following incubation, the solution was spun through a buffer exchange column (Thermo Scientific, 89882) to remove any excess dye.

#### Myelin Preparation and in vitro treatment

Myelin was prepared from mouse brains as previously described [19]. Purified myelin was passed through an insulin syringe prior to use to ensure cells were treated with a homogenous solution. Cells were treated with 100μg/ml myelin or vehicle control for 4 hours. For two replicates that included samples treated with CytoD, cells were pretreated for 30 minutes with 1μM CytoD (Millipore-Sigma, C8273) or DMSO and subsequently treated with 100μg/ml myelin or vehicle control (also containing CytoD or DMSO) for 4 hours. For the remaining two replicates that included samples treated with CytoD, no pretreatment was performed. Pretreatment with CytoD did not alter clusterin expression when compared to no pretreatment with CytoD, so all experiments were combined and plotted in Fig. 2F. Cells treated with myelin were washed once with PBS prior to RNA preparation.

#### Cytokine and H _2_ 0 _2_ in vitro treatment

OPCs were treated with 10ng/ml TNFα, 10ng/ml IFNγ, 10μM H_2_0_2_ or the relevant control for 3 hours and then processed for qPCR.

### ELISA

OPCs were grown as described above. A 10cm dish of approximately 2 million OPCs was treated with 3μM Aβ or an equivalent volume of vehicle for 72 hours. Cells were lysed for 15 minutes in RIPA buffer (PBS, 1% Triton X-100, 0.5% deoxycholic acid, 1% sodium dodecyl sulfate) supplemented with 1x protease inhibitor (MedChem Express HY-K0010). The insoluble material was removed by spinning the lysate at 15,000g for 15 minutes. The resulting lysate was used to quantify the amount of clusterin present in each sample using the Mouse Clusterin ELISA Kit (Thermo Fisher EM18RB) according to manufacturer’s instructions.

### Luminex Assay

OPCs were grown as described above. Cells were treated with 8μg/ml clusterin for 24 hours (1 replicate) or 72 hours (1 replicate). Media was then collected and spun to remove all cellular debris. Media was concentrated using a 3KD cutoff concentrator column (Thermo Fisher 88526). The resulting concentrate was analyzed using the Milliplex 32-plex Mouse Cytokine/Chemokine Panel (Millipore Sigma MCYTMAG-70K-Px32) according to manufacturer’s instructions.

#### Apoptotic cell preparation and in vitro treatment

To create apoptotic cells, Jurkats were treated with 150mJ of UV energy and incubated for 2–4 hours at 37°C in complete media consisting of RPMI 1640 Media (Gibco, 11875101), 10% FBS (R&D Systems, S12450H), and Pen-Strep (Gibco, 15140122). Apoptotic cells were washed and added to cultured OPCs at approximately a 1:1 ratio for 6 hours. OPCs were washed once prior to RNA isolation.

### Aβ Injections

C57BL/6J mice (8–14 weeks) were injected with CypHer-Aβ (ipsilateral) and NHS-Fluorescein (contralateral, Thermo Fisher 46410) as previously described [20]. Briefly, mice were anesthetized with a mixture of ketamine and xylazine and a small burr hole was drilled in the skull. 1μL of 100μM Aβ was injected at a speed of 200nL/minute into the right hemisphere. 1μL of 200μM NHS-Fluorescein diluted in PBS was injected into the left hemisphere at the same speed. Injections were targeted for 2mM lateral, 0mM anterior, and - 1.5mM deep relative to bregma. Mice were given ketoprofen following surgery and were euthanized 12 hours post-injection.

#### Immunofluorescence and electron microscopy.

Mice were deeply anesthetized with pentobarbitol and subsequently perfused with 5 units/mL heparin in saline followed by 10% buffered formalin, each for approximately one minute. For brain tissue, brains were rapidly dissected and post-fixed in 10% buffered formalin overnight at 4°C. Tissue was then transferred into 30% sucrose in PBS and allowed to sink for at least 24 hours. Brains were frozen in OCT, sectioned, and stored in PBS plus 0.02% NaAz until further staining.

Tissue or cultured cells were blocked with PBS, 1% BSA, 0.5% Triton-X 100, 2% normal donkey serum, and 1:200 CD16/CD32 (14-0161-82, 1:200, eBioscience) for at least one hour at room temperature. For stains utilizing a mouse primary antibody, tissue was blocked in Mouse on Mouse Blocking Reagent (MKB-2213, Vector Laboratories) according to manufacturer’s instructions for at least 1 hour at room temperature. Samples were incubated in primary antibodies overnight at 4°C with gentle agitation. Samples were then washed three times in TBS containing 0.3% Triton-X 100 and incubated in secondary antibodies overnight at 4°C with gentle agitation. Following secondary incubation, samples were stained with Hoechst (1:700, ThermoFisher Scientific, H3570) for 10 minutes at room temperature, washed three times in TBS containing 0.3% Triton-X 100, and mounted on slides using Aqua Mount Slide Mounting Media (Lerner Laboratories). Images were collected on a Leica TCS SP8 confocal microscope and processed using Fiji.

For quantitative ultrastructural analysis, mice were deeply anesthetized and transcardially perfused with 0.9% NaCl followed by a Millonig’s buffer solution (pH 7.3) containing 5% glutaraldehyde and 4% paraformaldehyde. Following the post-fixation, brains were harvested, vibratome sectioned and processed for standard electron microscopic analysis as previously described [21]. Briefly sagittal brain sections, containing the corpus callosum at the level of the fornix, were fixed in 1% cacodylate buffered osmium tetroxide, dehydrated in graded ice-cold ethanol and embedded in PolyBed 812 embedding resin (Polysciences, Inc., Fort Washington, PA). Ultrathin (70nm) sections were stained with uranyl acetate and lead citrate and imaged using a JEOL JEM 1400Plus transmission electron microscope (JEOL, Peabody, MA) equipped with a Gatan OneView CMOS camera (Gatan Inc., Pleasanton, CA). Electron micrographs were collected at 10,000X; a minimum of 100 myelinated axons per mouse were used to calculate g-ratios.

### Antibodies for Immunofluorescence

Primary antibodies used for immunofluorescence were PDGFRa (1:200, R&D Systems, AF1062), Olig2 (1:200, Millipore, MABN50), Clusterin (1:250, Abcam, AB184100), MBP (1:500, Abcam, ab7349) and Aβ (1:300, Cell Signaling Technology, 8243S). Secondary antibodies used were Donkey anti-Goat Cy3 2μg/mL, Jackson ImmunoResearch, 705-165-147), Donkey anti-Mouse 647 2μ g/mL, Jackson ImmunoResearch, 715-605-150), Donkey anti-Mouse 546 2μg/mL, Life Technologies, A10036), Donkey anti-Chicken 488 2μg/mL, Jackson ImmunoResearch, 703-545-155), Donkey anti-Goat 488 2μg/ml, Jackson ImmunoResearch, 705-545-147), Donkey anti-Rabbit Cy3 2μg/ml, Jackson ImmunoResearch, 711-165-152), Donkey anti-Rabbit 647 2μg/ml, Jackson ImmunoResearch, 711-605-152), and Donkey anti-Goat 647 2μg/mL, Invitrogen, A21447).

### Flow Cytometry

OPCs were incubated with 3μM CypHer-Aβ with 8μg/ml clusterin, 1μM CytoD, or a PBS vehicle control for 90 minutes. Cells were removed from the plate with 0.25% Trypsin-EDTA (Gibco 25200056), washed, and stained with Ghost Dye Violet 510 (0.5 μL/test, Tonbo biosciences, 13–0870). Cells were analyzed using a 3 laser, 10-color Gallios flow cytometer (Beckman-Coulter).

### Multiplex RNAscope (Human)

Human tissue was embedded in paraffin and cut into 15μM sections. Slices were heated for 48 hours to allow attachment. Tissue was subsequently processed using the V2 RNAscope Fluorescent Multiplex Reagent Kit (Advanced Cell Diagnostics, 323100) according to manufacturer’s instructions. Briefly, tissue was dehydrated using xylene and ethanol wash, treated with H_2_0_2_, and then incubated in Target Retrieval Buffer at 95°C for 15 minutes. Tissue was incubated with the supplied Protese Plus regent for 30 minutes. Target probes were hybridized to the tissue for two hours at 40°C, followed by hybridization of AMP1-FL (30 minutes, 40°C), AMP2-FL (15 minutes, 40°C), AMP3-FL (30 minutes, 40°C), and AMP4-FL (15 minutes, 40°C). Tissue was then treated with an HRP reagent for a single probe, followed by a unique secondary, and an HRP blocker. This process was repeated for each probe used. Samples were counterstained with supplied DAPI or Hoechst 33342 (1:700, ThermoFisher Scientific, H3570) and mounted on slides using ProLong Glass Antifade Mountant (ThermoFisher, P36980). The following target probes were used: Human *OLIG2*(Advanced Cell Diagnostics, 424191-C2), Human *PDGFRA* (Advanced Cell Diagnostics, 604481), Human *CLU*(Advanced Cell Diagnostics, 584771-C3), and the RNAscope 4-plex Negative Control Probes (Advanced Cell Diagnostics, 321831). The following dyes were used: Opal 520 (Akoya Biosciences, FP1487001), Opal 690 (Akoya Biosciences, FP1497001), and Opal 620 (Akoya Biosciences, FP1495001). Sections were imaged using a Leica TCS SP8 confocal microscope.

### Immunohistochemistry (Human)

5 μm thick tissue sections of formalin fixed, paraffin embedded (FFPE) brain tissue specimens were rehydrated in Xylene and descending alcohol series and heat-induced epitope retrieval (HIER) was performed in a pressure cooker (Tintoretriever, Bio SB) for 15 min at high pressure in a 0.05% Tween-20 solution. Endogenous peroxidase was quenched by incubation of sections in 1.5% hydrogen peroxide/0.005% Triton-X-100 diluted in pH 7.4 sterile phosphate buffered saline (PBS) (Invitrogen) for 20 min, following multiple washes in tap water and subsequently, 0.1 M Tris, pH 7.6. Non-specific antibody binding was minimized with sections incubated in 2% FBS/0.1 M Tris, pH 7.6. Clusterin (Proteintech) primary antibody was diluted in 2% FBS/0.1 M Tris, pH 7.6 at a dilution of 1:500. Sections were incubated with primary antibody over night at 4°C, washed one time in 0.1 M Tris, pH 7.6, followed by 2% FBS/0.1 M Tris, pH 7.6 for 5 min, incubated in goat ant-rabbit IgG HRP Conjugated secondary antibody (Millipore Sigma) for 1 hour, additionally washed one time in 0.1 M Tris, pH 7.6, followed by 2% FBS/0.1 M Tris, pH 7.6 for 5 min, and incubated in VectaStain ABC Peroxidase HRP Kit (diluted in 2% FBS/0.1 M Tris, pH 7.6 at 1:1000) for 1 hour. After a final wash in 0.1 M Tris, pH 7.6 for 5 min, immunocomplexes were visualized using the Vector Laboratories ImmPACT DAB Peroxidase (HRP) 3,3’-diaminobenzidine. Tissue sections were counterstained with hematoxylin (Sigma Aldrich, St. Louis, MO) for 2 minutes, dehydrated in ascending alcohol series and Xylene and cover slipped using Cytoseal 60 mounting medium (Thermo Scientific). For analysis of stains, slides of frontal cortex specimens stained with Clusterin antibodies were scanned on an Aperio AT2 scanner (Leica biosystems) at 20x magnification and digital slides analyzed using the QuPath platform (version 0.3.1, https://QuPath.github.io/, PMID: 29203879) on a Dell PC (Intel^®^ Xeon^®^ W-1270 CPU @ 3.40GHz/ 64 GB RAM/ 1 TB SSD Hard Drive) running Windows 10. Cortex and white matter were annotated for regional analysis. After exclusion of tissue and staining artifacts we used the ‘Positive Pixel Detection’ tool (Downsample factor 2, Gaussian sigma 1 μm, Hematoxylin threshold (‘Negative’) 1.5 OD units, DAB threshold (‘Positive’) 0.5 OD units) to determine the percentage of area covered by Clusterin staining.

### Combined in situ Hybridization and Immunohistochemistry (Human)

For in-situ hybridization, 5μm thick paraffin-embedded tissue sections on slides were rehydrated in xylene and series of ethanol solutions (100%, 90%, and 70%). Following air drying for 5 min at RT, slides were incubated with RNAscope^®^ Hydrogen peroxide for 10 min at RT, followed by 3 washing steps in distilled water. Antigen retrieval was performed in a steam bath for 15 min using RNAscope^®^ 1x target retrieval reagent. After a rinse in distilled water and incubation in 10% ethanol for 3 min slides were air dried at 60°C. Subsequently, slides were incubated with RNAscope^®^ Protease plus reagent for 30 min at 40°C in a HybEZ^™^ oven, followed by 3 washes in distilled water. Slides were then incubated with the following RNAscope^®^ probe for 2 hours at 40°C in a HybEZ^™^ oven: Hs-Clusterin (cat. number 606241). Following washes with 1X Wash buffer, slides were incubated with RNAscope^®^AMP1 solution for 30 min at 40°C. Subsequent incubations with other RNAscope^®^ AMP solutions, followed each by two washes with 1x RNAscope^®^ Wash buffer were completed as follows: AMP2–15 min at 40°C; AMP3–30 min at 40°C; AMP4–15 min at 40°C; AMP5–30 min at RT and AMP6–15 min at RT. After two washes in 1x RNAscope^®^ wash buffer slides were incubated in RNAscope^®^ Fast RED-B and RED-A mixture (1:60 ratio) for 10 min at RT, followed by two washes in tap water. For in situ hybridization/immunohistochemistry double labeling, sections were incubated in 2% FBS/0.1 M Tris, pH 7.6 for 5 min following RNAscope^®^ Fast RED incubation and two washes in tap water. Primary antibodies were diluted in 2% FBS/0.1 M Tris, pH 7.6 at the following dilutions: Ab5 (PMID: 16341263), 1:1000. Sections were incubated with primary antibody over night at 4°C, washed two times in 0.1 M Tris, pH 7.6 for 5 min each and incubated with biotinylated secondary antibody (Vector Laboratories; Burlingame, CA) diluted in 2% FBS/0.1 M Tris, pH 7.6 for 1 hour at room temperature. An avidin-biotin complex (ABC) system (Vectastain ABC Elite kit; Vector Laboratories, Burlingame, CA) was used to enhance detection of the immunocomplexes, which were visualized using the chromogen 3,3’-diaminobenzidine (DAB kit; KPL, Gaithersburg, MD). Tissue sections were counterstained with hematoxylin (Sigma Aldrich, St. Louis, MO), air dried at 60°C for 30 min and cover slipped using EcoMount^™^ mounting medium (Biocare Medical).

### RT-qPCR

RNA was extracted from samples using the ISOLATE II RNA Micro Kit (Bioline, BIO-52075) or the ISOLATE II RNA Mini Kit (Bioline, BIO-52073). Isolated RNA was reverse transcribed using the SensiFAST cDNA Synthesis Kit (Bioline, BIO-65054) or the iScript cDNA Synthesis Kit (Bio-Rad, 1708891). RT-qPCR was performed using the SensiFAST Probe No-ROX Kit (Bioline, BIO-86005) and TaqMan probes for *Gapdh* (ThermoFisher, Mm99999915_g1), *Mbp* (ThermoFisher, Mm01266402_m1 and Mm01266403_m1), *Plp1* (ThermoFisher, Mm00456892_m1), *Cnp* (ThermoFisher, Mm01306641_m1), *Myrf* (ThermoFisher, Mm01194959_m1), and *Clu* (ThermoFisher, Mm00442773_m1). Additionally, the SensiFAST SYBR No-ROX Kit (Bioline, BIO-98005) was used with primers for *Plp1* (Forward: GCCCCTACCAGACATCTAGC, Reverse: AGTCAGCCGCAAAACAGACT) and *Myrf* (CGGCGTCTCGACAGCCTCAA, Reverse: GACACGGCAAGAGAGCCGTCA). Data was collected using the CFX384 Real-Time System (Bio-rad).

### Statistical Analysis

Statistical analysis of all data was done using Prism 9 (Graphpad software). Significance was set at p< 0.05.

## Results

### Clusterin is upregulated by OPCs in 5xFAD and AD.

**Figure 1:**
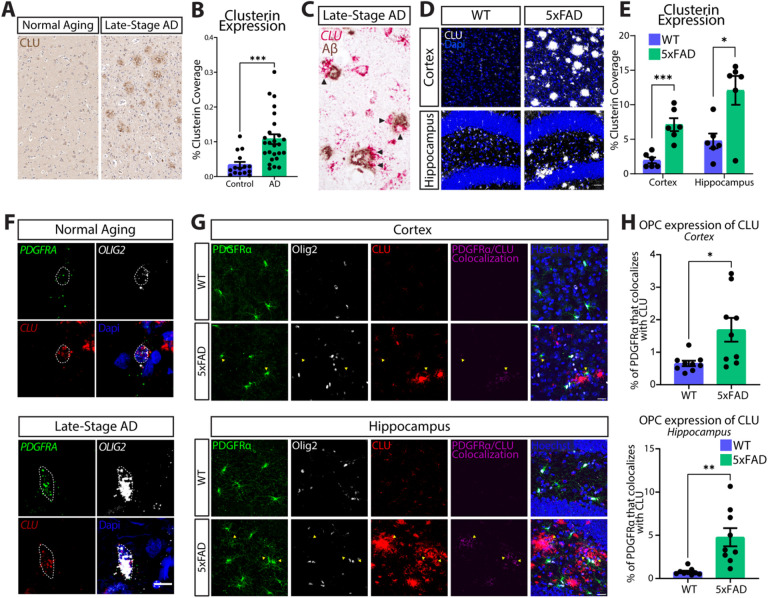
OPCs expresses the AD-risk factor clusterin. **A**, Representative image of Clusterin expression (immunohistochemistry, brown) in the cortex from a normal aging patient and a late-stage AD patient **B**, Quantification of clusterin coverage in the cortex of normal aging (*n*=15) and AD patients (*n*=26, from two independent experiments, depicted in A). Data analyzed using an unpaired t-test; t(39)=3.767. **C**, Detection of clusterin RNA (in situ hybridization, red) and Aβ protein (immunohistochemistry, brown) in late-stage AD brain (late-stage AD *n*=2; from two independent experiment). Arrowheads indicate clusterin-expressing cells around plaques. **D**, Representative images of clusterin expression (white) in the cortex and hippocampus of WT and 5xFAD mice. Scale bar=30μm. **E**, Quantification of clusterin coverage in the cortex and hippocampus of WT and 5xFAD mice (depicted in C; WT *n*=6, 5xFAD *n*=6; from two independent experiments). Statistics calculated using an unpaired Student’s t-test. Cortex: t(10)=5.049; Hippocampus: t(10)=3.119. **F**, *In situ hybridization* for OPCs *(PDGFRAin* green, *OLIG2in* white) expressing Clusterin *(CLU;* red) in normal aging and late-stage AD brains (normal aging *n*=1, late-stage AD *n*=1; from one independent experiment). Scale bar=10μm. **G**, Representative images of clusterin expression (red) in OPCs (Pdgfra; green and Olig2; white) in the cortex and hippocampus of WT and 5xFAD mice. Colocalization of Pdgfra and clusterin depicted in purple. Scale bar=20μm. **H**, quantification of the percentage of Pdgfra that colocalizes with clusterin in the cortex and hippocampus of WT and 5xFAD mice (depicted in **G**; WT *n*=9, 5xFAD *n*=9; from two independent experiments). Statistics calculated using an unpaired Student’s t-test. Cortex: t(16)=2.761; Hippocampus: t(16)=3.765.

Clusterin is upregulated in the brains of patients with Alzheimer’s disease and is a significant risk factor for Late-Stage AD [22]. Recent transcriptional analysis has shown that OPCs express clusterin in mice and humans but the function in this cell type is unknown [14, 15]. Here, we investigated the potential role of clusterin in OPCs. We confirmed that clusterin expression is increased in late-stage AD patients vs normal aging controls (Fig. 1A–B). We found that cells expressing clusterin RNA could be found directly surrounding Aβ plaques (Fig. 1C). This observation is conserved in pre-clinical models of AD, as clusterin is also found to be upregulated in multiple brain regions of the 5xFAD mouse model of AD (Fig. 1D–E). Because of the emerging role of OPCs and myelin in AD pathology, we investigated whether OPCs expressed clusterin in AD [5, 23]. We found that OPCs express clusterin both in the AD brain, as well as in normal aging (Fig. 1F). To further profile the OPC expression of clusterin in AD pathology, we quantified the expression of clusterin in OPCs in 5xFAD mice. We found that OPCs upregulate clusterin protein in 5xFAD mice compared to wildtype mice (Fig. 1G–H), mirroring human sequencing data demonstrating that OPCs upregulate clusterin RNA production in AD patients [15].

This data demonstrates that clusterin, a risk factor for late-onset AD, is upregulated in the parenchyma of AD patients and can be found in cells surrounding Aβ plaques. Furthermore, this data shows that human OPCs express clusterin in normal aging as well as AD, and that OPCs upregulate clusterin production in 5xFAD.

### Phagocytosis of oligomeric Aβ and cellular debris results in an upregulation of clusterin expression by OPCs.

**Figure 2:**
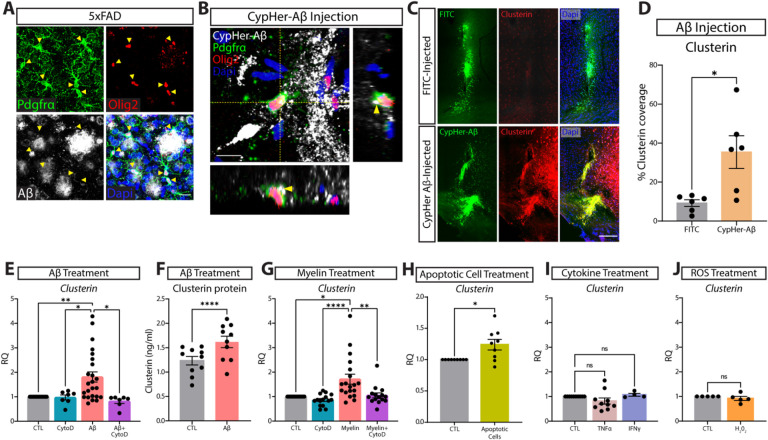
Phagocytosis of extracellular debris drives clusterin expression in OPCs. **A**, Representative image of OPCs (PDGFRa in green, Olig2 in red) surrounding Aβ plaques (white). Yellow arrowheads indicate OPCs that are extending processes into areas of Aβ accumulation (*n*=4 mice; from one independent experiment). Scale bar=20μm. **B**, Representative orthogonal view of CyPher-labeled Aβ (white) inside an OPC (PDGFRa in green, Olig2 in red) following intra-parenchymal injection of Aβ (*n*=6 mice; from one independent experiment). Yellow arrowheads indicate Aβ that can be seen inside the cell body of an OPC. Scale bar=10μm. **C**, Representative images of clusterin expression (red) in the ipsilateral (CypHer-Aβ injected; green) and contralateral (FITC injected; green) hemispheres following intra-parenchymal injection. Scale bar=100μm. **D**, Quantification of clusterin expression in the ipsilateral (Aβ-injected) and contralateral (FITC-injected) hemispheres following intra-parenchymal injection. (n=6 mice). Statistics calculated using a paired Student’s t-test; t(5)=2.979. **E**, qPCR analysis of clusterin expression in OPCs following a 4-hour *in vitro* treatment with 3μm Aβ and the phagocytosis blocker CytoD (1μm) or vehicle control (CTL *n*=24, CytoD *n*=7, Aβ n=24, Aβ+CytoD *n*=7; from seven independent experiments). Statistics calculated using a mixed effects analysis with a Tukey’s post-hoc analysis; F(1.121, 13.08) = 8.544. **F**, Quantification of clusterin protein in OPCs following 72-hour treatment with 3μM Aβ or vehicle control (CTL *n*=10, Aβ *n*=10, from two independent experiments). Statistics calculated using a paired Student’s t-test; t(9)=7.596. **G**, qPCR analysis of clusterin expression in OPCs following a 4-hour *in vitro* treatment with 100μg/ml myelin and CytoD (1μm) or vehicle control (CTL *n*=19, CytoD *n*=15, Myelin *n*=19, Myelin+CytoD *n*=15; from five independent experiments). Statistics calculated using a mixed effects analysis with a Tukey’s post-hoc analysis; F (1.389, 21.29) = 10.75. **H**, qPCR analysis of clusterin expression in OPCs following a 6-hour *in vitro* treatment with apoptotic cells (CTL *n*=9, Apoptotic cells *n*=9; from two independent experiments). Statistics calculated using a paired Student’s t-test; t(8)=2.835. **I**, qPCR analysis of clusterin expression in OPCs following a 3-hour *in vitro* treatment with 10ng/ml TNFα or 10ng/ml IFNγ (CTL *n*=10, TNFα *n*=10, IFNγ *n*=4; from 2 independent TNFα experiments or 1 independent IFNγ experiment). Statistics calculated using a mixed effects analysis; F (1.106, 11.61) = 1.897. **J**, qPCR analysis of clusterin expression in OPCs following a 3-hour *in vitro* treatment with 10μm H_2_0_2_ (*n*=5; from one independent experiment). Statistics calculated using a paired Student’s t-test; t(8)=0.3417. *p<0.05, **p<0.01, ****p<0.0001, ns=not significant. All error bars represent SEM.

Given our data showing that OPCs express the AD-risk factor clusterin, we wanted to determine if OPCs could contribute to AD pathology and interact with Aβ plaques. Using 5xFAD mice, we found that OPCs were surrounding and extending their processes into Aβ plaques (Fig. 2A). Because clusterin is known to facilitate debris clearance, we next wondered if OPCs were involved in the engulfment of Aβ in the brain[12]. To test this, we injected wildtype (WT) mice with Aβ oligomers labeled with CypHer5e, a dye that only fluoresces when it has entered the acidic environment of the lysosome. We found that OPCs around the injection site phagocytosed Aβ within 12 hours of injection (Fig. 2B, Supplementary Fig. 1).We also found that injecting Aβ was sufficient to drive an upregulation of clusterin expression in the brain 12 h after injection (Fig. 2C–D).

We next investigated whether OPCs could contribute to this Aβ-induced upregulation of clusterin. We treated primary OPCs *in vitro* with Aβ oligomers for 4 hours and observed a rapid increase in clusterin transcript expression (Fig. 2E). We subsequently used an ELISA assay to confirm that OPCs also upregulate the production of clusterin protein following treatment with Aβ (Fig. 2F). We found that phagocytosis of Aβ was necessary to drive this upregulation of clusterin, as treatment with cytochalasin D (CytoD), an actin polymerization inhibitor that blocks phagocytosis, prevented Aβ from increasing clusterin production in OPCs (Fig. 2E)[24–26]. Interestingly, we found that these changes in clusterin were not specific to the phagocytic clearance of Aβ oligomers, but were rather generalizable to instances in which OPCs engulfed large debris. We found that treatment of OPCs with myelin debris and apoptotic cells also produced a similar upregulation of clusterin that was abrogated by exposure to CytoD (Fig. 2G–H). In order to eliminate the possibility that this clusterin upregulation was simply a response to any cellular stressor present in AD, we treated OPCs with other factors known to be upregulated in Alzheimer’s disease, including TNFα, IFNγ, and reactive oxygen species (ROS), which all failed to produce any change in clusterin expression (Fig. 2I–J)[27–29].

### Clusterin does not promote the phagocytosis of oligomeric Aβ.

**Figure 3:**
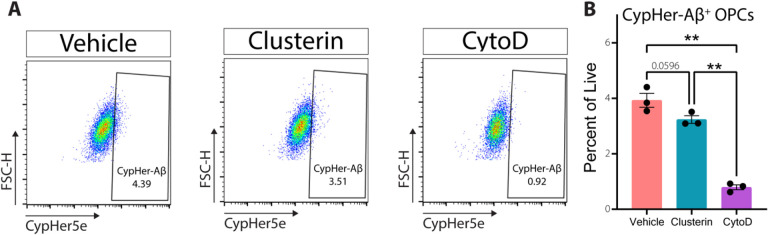
Clusterin does not alter OPC phagocytosis of oligomeric Aβ. **A**, Representative flow gating (following singlets/singlets/live gates) of OPCs incubated for 90 minutes with 3μm CypHer5e-labeled Aβ oligomers (all conditions) with the addition of 8μg/ml clusterin or CytoD (Vehicle n=3, Clusterin *n*=3, CytoD *n*=3; from one independent experiment). CypHer+ gate was drawn so that less than 1% of cells in the CytoD samples fell within the positive gate. **B**, Quantification of OPCs staining positive for CypHer-Aβ as depicted in **A**. Statistics calculated using a repeated measures one-way ANOVA with a Tukey’s post-hoc analysis; F(2,4)= 293.3. **p<0.01. All error bars represent SEM.

In previous studies, clusterin has been shown to increase the phagocytic capacity of both professional and non-professional phagocytes alike[24, 25]. This led us to investigate whether clusterin altered the kinetics of phagocytosis in OPCs. The addition of exogenous clusterin did not change the ability of OPCs to engulf Aβ *in vitro* (Fig. 3A–B), indicating that phagocytosis regulates clusterin expression in OPCs, but clusterin does not subsequently regulate the phagocytosis of Aβ. In sum, these results indicate that OPCs can clear extracellular debris, including Aβ, and that phagocytosis of protein or cellular debris is responsible for driving clusterin expression in OPCs.

### Exogenous clusterin inhibits OPC differentiation.

**Figure 4:**
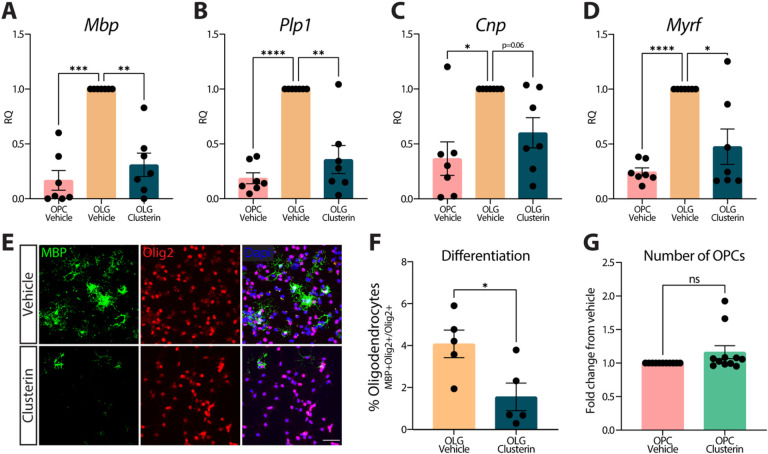
Exogenous clusterin inhibits OPC differentiation. Expression of *Mbp* (**A**), *Plp1* (**B**), Cnp(**C**), and *Myrf* (**D**), measured by qPCR in OPCs cultured in proliferation media (OPC Vehicle), differentiation media (OLG Vehicle), or differentiation media supplemented with 8μg/ml of clusterin (OLG Clusterin) for 72 hours (n=7 for all conditions; from 3 independent experiments). Statistics calculated using a repeated measures one-way ANOVA with a Tukey’s post-hoc analysis; *Mbp* F(2,6)=27.41, *Plp1* F(2,6)=25.72, *Cnp*F(2,6)=9.776, *Myf* F(2,6)=16.41. **E**, Representative images of OPCs cultured in differentiation media with or without 8μg/ml of clusterin for 72 hours and stained for oligodendrocyte markers (MBP in green, Olig2 in red). Scale bar= 50μm. **F**, Quantification of the number of OPCs that differentiated in to oligodendrocytes following treatment with clusterin (depicted in **E**; *n*=5 for all conditions; from two independent experiments). Statistics calculated using a paired Student’s t-test; t(4)=2.780. **G**, Quantification of the number of OPCs present using a Cell Counting Kit-8 assay following a 72 hour incubation in proliferation media with or without 8μg/ml clusterin (*n*=11 for all conditions; from four independent experiments). *p<0.05, **p<0.01, ****p<0.0001, ns=not significant. All error bars represent SEM.

The formation of new myelin has been increasingly recognized as a critical component of memory function [3, 4]. In fact, a recent study demonstrated that drugs promoting nascent myelin formation results in improved memory performance in a model of AD, indicating the importance of understanding what factors are preventing the differentiation of OPCs into oligodendrocytes[5]. A multitude of studies have demonstrated that cellular debris and protein aggregation prevent OPC differentiation, although the mechanism by which this occurs remains unclear[30, 31]. This data, along with our observation that debris clearance drives clusterin expression in OPCs (Fig. 2E–H), made us question whether clusterin might be inhibiting OPC differentiation. Consequently, we treated differentiating OPCs with exogenous clusterin and observed a striking decrease not only in genes encoding myelin proteins *(Mbp, Plp1, Cnp),* but also in *Myrf,* the master transcriptional regulator of the OPC differentiation program 72 hours after treatment (Fig. 4A–D). We subsequently observed fewer MBP-positive oligodendrocytes when OPCs were differentiated for 72 hours in the presence of clusterin compared to clusterin-free media (Fig. 4E–F). Importantly, this decrease in differentiation is not due to OPC death, as there was no difference in OPC number following clusterin treatment (Fig. 4G). Overall, this data shows that clusterin is regulated by the phagocytosis of debris and is a potent inhibitor of OPC differentiation.

### Clusterin inhibits differentiation by reducing IL-9 production.

**Figure 5:**
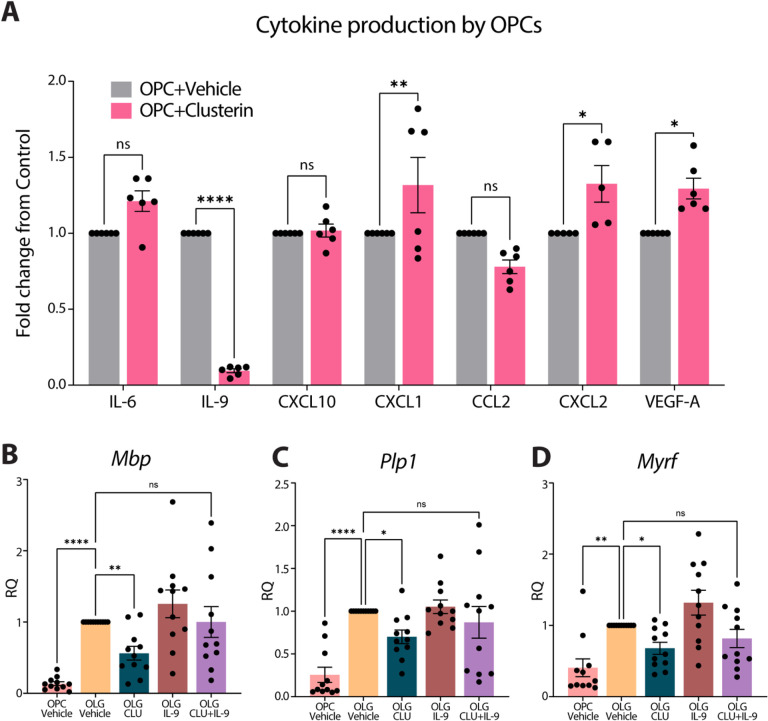
Clusterin inhibits differentiation by blocking IL-9 production. **A**, Quantification of cytokines present in the supernatant from OPCs treated with 8μg/mL clusterin or vehicle control (*n*=6 biological replicates for each condition, from two independent experiments). Data analyzed using a two-way repeated measures ANOVA with a Sidak’s multiple comparison post-hoc analysis, F (6, 34) = 23.93. Expression of *Mbp* (**B**), *Plp1* (**C**), and *Myrf* (**D**), measured by qPCR in OPCs cultured in proliferation media (OPC Vehicle), differentiation media (OLG Vehicle), differentiation media supplemented with 8μg/ml of clusterin (OLG CLU), differentiation media supplemented with 100ng/ml IL-9 (OLG IL-9), or differentiation media supplemented with 8μg/ml of clusterin and 100ng/ml IL-9 (OLG CLU+IL-9) for 72 hours (*n*=11 for all conditions; from 3 independent experiments). Statistics calculated using a repeated measures one-way ANOVA with a Tukey’s post-hoc analysis; *Mbp* F(10,40)=10.15, *Plp1* F(10,40)=11.96, *Myrf* F(10,40)=10.32. *p<0.05, **p<0.01, ****p<0.0001, ns=not significant. All error bars represent SEM.

We next investigated what factors might be mediating clusterin’s inhibition of OPC differentiation. OPCs have been shown to produce a variety of growth factors and cytokines that can significantly alter their local environment [32–36]. Additionally, clusterin has been shown to regulate to production of cytokines [37–39]. Based on this data, we wondered if clusterin might be inhibiting OPC differentiation by affecting growth factor and cytokine production. We performed a Luminex Assay on the supernatant of OPCs treated with vehicle or clusterin and found that clusterin altered the secretion of multiple proteins, mostly notably increasing the production of VEGF and significantly decreasing the production of IL-9 (Fig. 5A). Since growth factors from the VEGF family have been shown to induce OPC proliferation [40], we tested whether the increase in VEGF following clusterin treatment was responsible for keeping OPCs in an undifferentiated state. However, treatment of OPCs with a combination of clusterin and an anti-VEGF antibody failed to rescue the differentiation defect observed with clusterin treatment (Supplementary Fig. 2A-C).

The next candidate we assessed as the potential mechanism for the effects of clusterin on OPCs was IL-9, since its production was reduced by more than 90% following clusterin treatment (Fig. 5A). IL-9 is a relatively understudied cytokine known to be produced by T-cells[41]. Surprisingly, we found that the addition of exogenous IL-9 was sufficient to rescue the differentiation deficits induced by clusterin treatment (Fig. 5B–D). Overall, this data demonstrates that clusterin blocks the differentiation of OPCs by inhibiting the production of IL-9, and that IL-9 is an important factor in proper differentiation of OPCs.

#### Clusterin deletion improves myelination in vivo.

**Figure 6:**
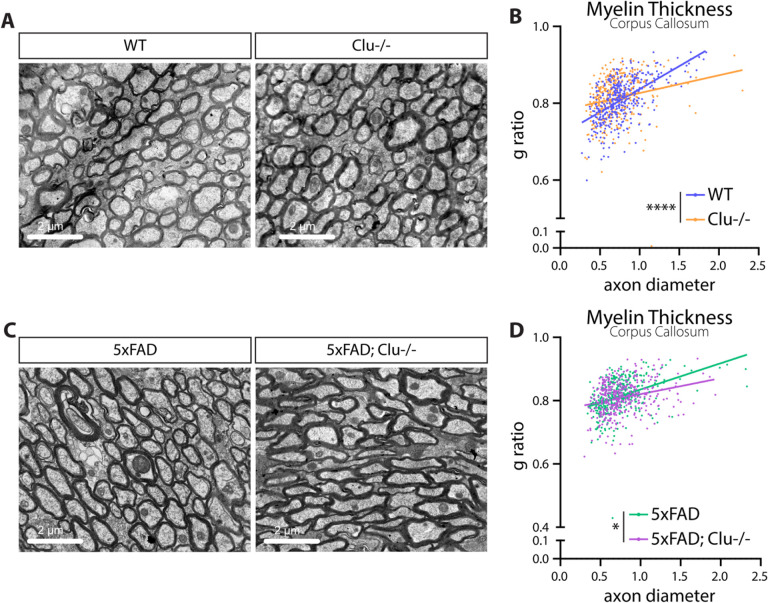
Deletion of clusterin improves myelination in WT and 5XFAD mice. **A**, Representative electron microscopy images of myelinated axons from the corpus callosum of 9-month old WT and clusterin knockout (Clu^−/−^) mice. **B**, Quantification of the myelin g-ratio plotted against axon diameter in WT and Clu−/− mice. Lines represent linear regression of the plotted data and p-values represent comparison of slopes. **C**, Representative electron microscopy images of myelinated axons from the corpus callosum of 9-month old 5xFAD and 5xFAD; Clu−/− mice. **B**, Quantification of the myelin g-ratio plotted against axon diameter in 5xFAD and 5xFAD; Clu−/− mice. Lines represent linear regression of the plotted data and p-values represent comparison of slopes. *p<0.05, ****p<0.0001.

Given our data showing that the expression of myelin genes was inhibited by clusterin, we next wanted to test if clusterin deletion would affect myelination in vivo. We performed transmission electron microscopy on the corpus callosum in 9-month-old clusterin knockout (Clu−/−) and WT mice. We measured the g-ratio of myelinated axons and found that Clu−/− mice had a lower g-ratio than control animals, indicating thicker myelination (Fig. 6A–B). This result shows that, at baseline, clusterin expression plays an inhibitory role in myelination. Since clusterin expression increases in the 5xFAD model, we next explored if deletion of clusterin could improve the myelination deficits observed in these mice [42–44]. To do this, we bred Clu−/− mice to 5xFAD mice to generate 5xFAD; Clu−/− mice. We again performed transmission electron microscopy and measured the g-ratio of myelinated axons in the corpus callosum at 9 months old. Just as with the WT mice, we observed a decrease in the g-ratio of myelinated axons in the 5xFAD; Clu−/− mice compared to the 5xFAD controls (Fig. 6C–D). Overall, our results indicate that clusterin serves as an inhibitor of myelination, both under homeostatic conditions and in the context of 5xFAD pathology.

## Discussion

Since the discovery of Aβ plaques in the brains of AD patients, therapeutic development has been focused on reducing plaque load. However, no therapeutic candidate, even when successful at reducing plaques, has succeeded in slowing disease progression[45]. Over recent years, a growing body of literature has speculated that other altered biological processes may be the driving force behind the clinical decline observed in AD patients [46]. Increasingly, OPCs and myelin have been implicated in the etiology and progression of AD symptoms. A recent study demonstrated that the ablation of senescent OPCs was sufficient to improve the memory impairment observed in the APP/PS1 model of AD[23]. Additionally, multiple studies have shown that inhibiting OPC differentiation prevents memory consolidation and recall, and that therapeutically increasing myelination can improve memory performance in AD models [3–5, 47]. Here, we offer a potential mechanism for the pervasive myelin deficits observed in Alzheimer’s disease and the memory decline associated with these deficits [48, 49]. We demonstrate that clusterin is upregulated in the brains of AD patients as well as in a mouse model of AD. We found that human OPCs express clusterin in both normal aging as well as AD patients, and that OPCs in 5xFAD mice upregulate the production of the clusterin protein. Phagocytosis of debris, including Aβ oligomers, myelin, and apoptotic cells, efficiently drives the upregulation of clusterin in OPCs. We show that clusterin, through its effects on the production of IL-9, is a potent inhibitor of OPC differentiation and the production of myelin proteins. Finally, we demonstrate that *in vivo* knockout of clusterin produces thicker myelin sheaths under normal conditions and abrogates myelin loss observed in 5xFAD mice.

A common single-nucleotide polymorphism (SNP) in clusterin has been recognized as a significant risk factor for late onset AD for over a decade [9]. While there are conflicting reports in the literature regarding how this SNP effects the function and accumulation of clusterin, there are multiple studies indicating that that increased levels of clusterin in the plasma of AD patients, regardless of the presence of a SNP at the CLU locus, correlates with a more rapid cognitive decline and an increase in brain atrophy [11, 50–52]. These correlative studies demonstrating that excess levels of clusterin are associated with more severe AD symptoms are supported by studies indicating that removing clusterin in mouse models of AD results in a reduced plaque load as well as improved performance on memory tasks [53–55].

There are, however, reports in the literature indicting that clusterin might be involved in the clearance of Aβ plaques and the protection of neurons [56, 57]. These discrepancies likely indicate that clusterin is a multi-functional protein that may be beneficial when expressed at homeostatic levels, but could become detrimental when significantly upregulated in the context of disease pathology. Additionally, since there is an increasing amount of data indicating that the reduction of Aβ plaques in AD patients does not improve clinical symptoms, it is possible that clusterin’s inhibition of OPC differentiation, as described here, may be one of the mechanism though which increased levels of clusterin might contribute to AD progression[58].

It remains to be determined exactly which cell types might be contributing to the increase in clusterin observed in Alzheimer’s disease. While we have demonstrated that OPCs upregulate clusterin production in response to the phagocytosis of Aβ as well as other cellular debris, and human single cell sequencing data indicates that OPCs are the only cell type in the brain that show an increase in clusterin expression in AD patients compared to healthy controls, it is known that astrocytes produce a significant amount of clusterin[15, 59]. However, while the exact cellular sources of elevated levels of clusterin in AD is still an open question, the fact that the most abundant isoform of clusterin encodes for a secreted chaperone protein indicates that clusterin from any cellular source, whether OPCs or any other cell type, can impact the function and differentiation capacity of OPCs[60].

We were surprised to find that clusterin decreased the differentiation of OPCs by blocking the production of IL-9. While IL9 receptor expression has been documented in OPCs, this is the first time that OPCs have been shown to produce IL-9 and that IL-9 is important for the proper differentiation of OPCs[61]. While T-cells are known to be the main producers of IL-9, there is increasing evidence in the literature that OPCs can also contribute to cytokine production [32, 34, 62, 63]. The ability of OPCs to function as immunomodulatory cells is garnering increasing support in the literature, and our data indicating the importance of IL-9 in the function of OPCs offers an intriguing avenue for additional investigation [64, 65].

It has been known that clusterin is increased in the brains of AD patients for over three decades [66]. However, the mechanism of clusterin’s effects on the progression of AD remains unclear. With an antisense-oligonucleotide targeting clusterin having progressed to Phase 3 clinical trials for prostate cancer, our data demonstrating that clusterin negatively affects OPCs and myelin production offers a new therapeutic avenue to potentially improve myelin integrity and memory deficits plaguing AD patients[67, 68]. What’s more, our data linking clusterin and myelin dysregulation is supported by MRI data demonstrating that the clusterin risk allele is associated with a decrease in white matter integrity in healthy young adults, prior to onset of any cognitive decline [69].

## Conclusions

Overall, our data offers a novel mechanism for the association between clusterin as a genetic risk factor and Alzheimer’s disease myelin pathology. Further, our study provides a foundation for development of therapeutic targeting Clusterin for the treatment of AD.

## Data Availability

The datasets and material generated during and/or analyzed during the current study are available from the corresponding author upon reasonable request.

## References

[1] Association As. 2021 Alzheimer’s disease facts and figures. Alzheimer’s Dement. 2021;17:327–406. 33756057 10.1002/alz.12328

[2] Roher AE, Weiss N, Kokjohn TA, Kuo Y-M, Kalback W, Anthony J, Watson D, Luehrs DC, Sue L, Walker D, Increased Aβ Peptides and Reduced Cholesterol and Myelin Proteins Characterize White Matter Degeneration in Alzheimer’s Disease. Biochemistry. 2002;41:11080–90. 12220172 10.1021/bi026173d

[3] Pan S, Mayoral SR, Choi HS, Chan JR, Kheirbek MA. Preservation of a remote fear memory requires new myelin formation. Nat Neurosci. 2020;23:487–99. 32042175 10.1038/s41593-019-0582-1 PMC7213814

[4] Steadman PE, Xia F, Ahmed M, Mocle AJ, Penning ARA, Geraghty AC, Steenland HW, Monje M, Josselyn SA, Frankland PW. Disruption of Oligodendrogenesis Impairs Memory Consolidation in Adult Mice. Neuron. 2020;105:150–e164156. 31753579 10.1016/j.neuron.2019.10.013 PMC7579726

[5] Chen J-F, Liu K, Hu B, Li R-R, Xin W, Chen H, Wang F, Chen L, Li R-X, Ren S-Y, Enhancing myelin renewal reverses cognitive dysfunction in a murine model of Alzheimer’s disease. Neuron. 2021;109:2292–e23072295. 34102111 10.1016/j.neuron.2021.05.012 PMC8298291

[6] Kang SH, Fukaya M, Yang JK, Rothstein JD, Bergles DE. NG2 + CNS glial progenitors remain committed to the oligodendrocyte lineage in postnatal life and following neurodegeneration. Neuron. 2010;68:668–81. 21092857 10.1016/j.neuron.2010.09.009 PMC2989827

[7] Dawson MR, Polito A, Levine JM, Reynolds R. NG2-expressing glial progenitor cells: an abundant and widespread population of cycling cells in the adult rat CNS. Mol Cell Neurosci. 2003;24:476–88. 14572468 10.1016/s1044-7431(03)00210-0

[8] McKenzie IA, Ohayon D, Li H, de Faria JP, Emery B, Tohyama K, Richardson WD. Motor skill learning requires active central myelination. Science. 2014;346:318–22. 25324381 10.1126/science.1254960 PMC6324726

[9] Harold D, Abraham R, Hollingworth P, Sims R, Gerrish A, Hamshere ML, Pahwa JS, Moskvina V, Dowzell K, Williams A, Genome-wide association study identifies variants at CLU and PICALM associated with Alzheimer’s disease. Nat Genet. 2009;41:1088–93. 19734902 10.1038/ng.440 PMC2845877

[10] Falgarone G, Chiocchia G. Chap. 8: Clusterin: A multifacet protein at the crossroad of inflammation and autoimmunity. Adv Cancer Res. 2009;104:139–70. 19878776 10.1016/S0065-230X(09)04008-1

[11] Thambisetty M, Simmons A, Velayudhan L, Hye A, Campbell J, Zhang Y, Wahlund LO, Westman E, Kinsey A, Güntert A, Association of plasma clusterin concentration with severity, pathology, and progression in Alzheimer disease. Arch Gen Psychiatry. 2010;67:739–48. 20603455 10.1001/archgenpsychiatry.2010.78 PMC3111021

[12] Wyatt AR, Yerbury JJ, Berghofer P, Greguric I, Katsifis A, Dobson CM, Wilson MR. Clusterin facilitates in vivo clearance of extracellular misfolded proteins. Cell Mol Life Sci. 2011;68:3919–31. 21505792 10.1007/s00018-011-0684-8 PMC11115182

[13] Loveless S, Neal JW, Howell OW, Harding KE, Sarkies P, Evans R, Bevan RJ, Hakobyan S, Harris CL, Robertson NP, Morgan BP. Tissue microarray methodology identifies complement pathway activation and dysregulation in progressive multiple sclerosis. Brain Pathol. 2018;28:507–20. 28707765 10.1111/bpa.12546 PMC8028318

[14] Beiter RM, Rivet-Noor C, Merchak AR, Bai R, Johanson DM, Slogar E, Sol-Church K, Overall CC, Gaultier A. Evidence for oligodendrocyte progenitor cell heterogeneity in the adult mouse brain. Sci Rep. 2022;12:12921. 35902669 10.1038/s41598-022-17081-7 PMC9334628

[15] Grubman A, Chew G, Ouyang JF, Sun G, Choo XY, McLean C, Simmons RK, Buckberry S, Vargas-Landin DB, Poppe D, A single-cell atlas of entorhinal cortex from individuals with Alzheimer’s disease reveals cell-type-specific gene expression regulation. Nat Neurosci. 2019;22:2087–97. 31768052 10.1038/s41593-019-0539-4

[16] Oakley H, Cole SL, Logan S, Maus E, Shao P, Craft J, Guillozet-Bongaarts A, Ohno M, Disterhoft J, Van Eldik L, Intraneuronal beta-amyloid aggregates, neurodegeneration, and neuron loss in transgenic mice with five familial Alzheimer’s disease mutations: potential factors in amyloid plaque formation. J Neurosci. 2006;26:10129–40. 17021169 10.1523/JNEUROSCI.1202-06.2006 PMC6674618

[17] Pedraza CE, Monk R, Lei J, Hao Q, Macklin WB. Production, characterization, and efficient transfection of highly pure oligodendrocyte precursor cultures from mouse embryonic neural progenitors. Glia. 2008;56:1339–52. 18512250 10.1002/glia.20702 PMC4395472

[18] Stine WB, Jungbauer L, Yu C, LaDu MJ. Preparing Synthetic Aβ in Different Aggregation States. In Alzheimer’s Disease and Frontotemporal Dementia: Methods and Protocols. Edited by Roberson ED. Totowa, NJ: Humana Press; 2011: 13–32. 10.1007/978-1-60761-744-0_2 PMC3752843 20967580

[19] Gaultier A, Wu X, Le Moan N, Takimoto S, Mukandala G, Akassoglou K, Campana WM, Gonias SL. Low-density lipoprotein receptor-related protein 1 is an essential receptor for myelin phagocytosis. J Cell Sci. 2009;122:1155–62. 19299462 10.1242/jcs.040717 PMC2714439

[20] Pluvinage JV, Haney MS, Smith BAH, Sun J, Iram T, Bonanno L, Li L, Lee DP, Morgens DW, Yang AC, CD22 blockade restores homeostatic microglial phagocytosis in ageing brains. Nature. 2019;568:187–92. 30944478 10.1038/s41586-019-1088-4 PMC6574119

[21] Fernandez-Castaneda A, Chappell MS, Rosen DA, Seki SM, Beiter RM, Johanson DM, Liskey D, Farber E, Onengut-Gumuscu S, Overall CC, The active contribution of OPCs to neuroinflammation is mediated by LRP1. Acta Neuropathol. 2020;139:365–82. 31552482 10.1007/s00401-019-02073-1 PMC6994364

[22] Herring SK, Moon H-J, Rawal P, Chhibber A, Zhao L. Brain clusterin protein isoforms and mitochondrial localization. eLife. 2019;8:e48255. 31738162 10.7554/eLife.48255 PMC6860991

[23] Zhang P, Kishimoto Y, Grammatikakis I, Gottimukkala K, Cutler RG, Zhang S, Abdelmohsen K, Bohr VA, Misra Sen J, Gorospe M, Mattson MP. Senolytic therapy alleviates Aβ-associated oligodendrocyte progenitor cell senescence and cognitive deficits in an Alzheimer’s disease model. Nat Neurosci 2019. 10.1038/s41593-019-0372-9 PMC6605052 30936558

[24] Weng X, Zhao H, Guan Q, Shi G, Feng S, Gleave ME, Nguan CCY, Du C. Clusterin regulates macrophage expansion, polarization and phagocytic activity in response to inflammation in the kidneys. Immunol Cell Biology. 2021;99:274–87. 10.1111/imcb.12405 PMC7984284 32935392

[25] Bartl MM, Luckenbach T, Bergner O, Ullrich O, Koch-Brandt C. Multiple receptors mediate apoJ-dependent clearance of cellular debris into nonprofessional phagocytes. Exp Cell Res. 2001;271:130–41. 11697889 10.1006/excr.2001.5358

[26] Ribes S, Ebert S, Regen T, Agarwal A, Tauber SC, Czesnik D, Spreer A, Bunkowski S, Eiffert H, Hanisch UK, Toll-like receptor stimulation enhances phagocytosis and intracellular killing of nonencapsulated and encapsulated Streptococcus pneumoniae by murine microglia. Infect Immun. 2010;78:865–71. 19933834 10.1128/IAI.01110-09 PMC2812218

[27] Browne TC, McQuillan K, McManus RM, O’Reilly J-A, Mills KHG, Lynch MA. IFN-γ Production by Amyloid β-Specific Th1 Cells Promotes Microglial Activation and Increases Plaque Burden in a Mouse Model of Alzheimer’s Disease. J Immunol. 2013;190:2241. 23365075 10.4049/jimmunol.1200947

[28] Bona DD, Candore G, Franceschi C, Licastro F, Colonna-Romano G, Cammà C, Lio D, Caruso C. Systematic review by meta-analyses on the possible role of TNF-α polymorphisms in association with Alzheimer’s disease. Brain Res Rev. 2009;61:60–8. 19445962 10.1016/j.brainresrev.2009.05.001

[29] Yang J, Zhang X, Yuan P, Yang J, Xu Y, Grutzendler J, Shao Y, Moore A, Ran C. Oxalate-curcumin–based probe for micro- and macroimaging of reactive oxygen species in Alzheimer’s disease. Proceedings of the National Academy of Sciences 2017, 114:12384. 10.1073/pnas.1706248114 PMC5703278 29109280

[30] Kotter MR, Li WW, Zhao C, Franklin RJ. Myelin impairs CNS remyelination by inhibiting oligodendrocyte precursor cell differentiation. J Neurosci. 2006;26:328–32. 16399703 10.1523/JNEUROSCI.2615-05.2006 PMC6674302

[31] Stoffels JMJ, de Jonge JC, Stancic M, Nomden A, van Strien ME, Ma D, Šišková Z, Maier O, ffrench-Constant C, Franklin RJM, Fibronectin aggregation in multiple sclerosis lesions impairs remyelination. Brain. 2013;136:116–31. 23365094 10.1093/brain/aws313

[32] Kang Z, Wang C, Zepp J, Wu L, Sun K, Zhao J, Chandrasekharan U, DiCorleto PE, Trapp BD, Ransohoff RM, Li X. Act1 mediates IL-17-induced EAE pathogenesis selectively in NG2 + glial cells. Nat Neurosci. 2013;16:1401–8. 23995070 10.1038/nn.3505 PMC4106025

[33] Wang C, Zhang C-J, Martin BN, Bulek K, Kang Z, Zhao J, Bian G, Carman JA, Gao J, Dongre A, IL-17 induced NOTCH1 activation in oligodendrocyte progenitor cells enhances proliferation and inflammatory gene expression. Nat Commun. 2017;8:15508. 28561022 10.1038/ncomms15508 PMC5460031

[34] Moyon S, Dubessy AL, Aigrot MS, Trotter M, Huang JK, Dauphinot L, Potier MC, Kerninon C, Melik Parsadaniantz S, Franklin RJ, Lubetzki C. Demyelination causes adult CNS progenitors to revert to an immature state and express immune cues that support their migration. J Neurosci. 2015;35:4–20. 25568099 10.1523/JNEUROSCI.0849-14.2015 PMC6605244

[35] Zhang YW, Denham J, Thies RS. Oligodendrocyte progenitor cells derived from human embryonic stem cells express neurotrophic factors. Stem Cells Dev. 2006;15:943–52. 17253955 10.1089/scd.2006.15.943

[36] Birey F, Kloc M, Chavali M, Hussein I, Wilson M, Christoffel DJ, Chen T, Frohman MA, Robinson JK, Russo SJ, Genetic and Stress-Induced Loss of NG2 Glia Triggers Emergence of Depressive-like Behaviors through Reduced Secretion of FGF2. Neuron. 2015;88:941–56. 26606998 10.1016/j.neuron.2015.10.046 PMC5354631

[37] Shim Y-J, Kang B-H, Choi B-K, Park I-S, Min B-H. Clusterin induces the secretion of TNF-α and the chemotactic migration of macrophages. Biochem Biophys Res Commun. 2012;422:200–5. 22575505 10.1016/j.bbrc.2012.04.162

[38] Liang Y, Liang N, Ma Y, Tang S, Ye S, Xiao F. Role of Clusterin/NF-KB in the secretion of senescence-associated secretory phenotype in Cr(VI)-induced premature senescent L-02 hepatocytes. Ecotoxicol Environ Saf. 2021;219:112343. 34020271 10.1016/j.ecoenv.2021.112343

[39] Shim YJ, Kang BH, Jeon HS, Park IS, Lee KU, Lee IK, Park GH, Lee KM, Schedin P, Min BH. Clusterin induces matrix metalloproteinase-9 expression via ERK1/2 and PI3K/Akt/NF-KB pathways in monocytes/macrophages. J Leukoc Biol. 2011;90:761–9. 21742938 10.1189/jlb.0311110

[40] Hiratsuka D, Kurganov E, Furube E, Morita M, Miyata S. VEGF- and PDGF-dependent proliferation of oligodendrocyte progenitor cells in the medulla oblongata after LPC-induced focal demyelination. J Neuroimmunol. 2019;332:176–86. 31075641 10.1016/j.jneuroim.2019.04.016

[41] Perumal NB, Kaplan MH. Regulating Il9 transcription in T helper cells. Trends Immunol. 2011;32:146–50. 21371941 10.1016/j.it.2011.01.006 PMC3070825

[42] Desai MK, Sudol KL, Janelsins MC, Mastrangelo MA, Frazer ME, Bowers WJ. Triple-transgenic Alzheimer’s disease mice exhibit region-specific abnormalities in brain myelination patterns prior to appearance of amyloid and tau pathology. Glia. 2009;57:54–65. 18661556 10.1002/glia.20734 PMC2584762

[43] Vanzulli I, Papanikolaou M, De-La-Rocha IC, Pieropan F, Rivera AD, Gomez-Nicola D, Verkhratsky A, Rodriguez JJ, Butt AM. Disruption of oligodendrocyte progenitor cells is an early sign of pathology in the triple transgenic mouse model of Alzheimer’s disease. Neurobiol Aging. 2020;94:130–9. 32619874 10.1016/j.neurobiolaging.2020.05.016 PMC7453384

[44] Wu D, Tang X, Gu LH, Li XL, Qi XY, Bai F, Chen XC, Wang JZ, Ren QG, Zhang ZJ. LINGO-1 antibody ameliorates myelin impairment and spatial memory deficits in the early stage of 5XFAD mice. CNS Neurosci Ther. 2018;24:381–93. 29427384 10.1111/cns.12809 PMC6489849

[45] Holmes C, Boche D, Wilkinson D, Yadegarfar G, Hopkins V, Bayer A, Jones RW, Bullock R, Love S, Neal JW, Long-term effects of Aβ42 immunisation in Alzheimer’s disease: follow-up of a randomised, placebo-controlled phase I trial. Lancet. 2008;372:216–23. 18640458 10.1016/S0140-6736(08)61075-2

[46] Mullane K, Williams M. Alzheimer’s disease beyond amyloid: Can the repetitive failures of amyloid-targeted therapeutics inform future approaches to dementia drug discovery? Biochem Pharmacol. 2020;177:113945. 32247851 10.1016/j.bcp.2020.113945

[47] Wang F, Ren S-Y, Chen J-F, Liu K, Li R-X, Li Z-F, Hu B, Niu J-Q, Xiao L, Chan JR, Mei F. Myelin degeneration and diminished myelin renewal contribute to age-related deficits in memory. Nat Neurosci. 2020;23:481–6. 32042174 10.1038/s41593-020-0588-8 PMC7306053

[48] Guo X, Wang Z, Li K, Li Z, Qi Z, Jin Z, Yao L, Chen K. Voxel-based assessment of gray and white matter volumes in Alzheimer’s disease. Neurosci Lett. 2010;468:146–50. 19879920 10.1016/j.neulet.2009.10.086 PMC2844895

[49] Zhan X, Jickling C, Ander GCP, Liu B, Stamova D, Cox B, Jin C, DeCarli L-W, Sharp CR. Myelin Injury and Degraded Myelin Vesicles in Alzheimer’s Disease. Curr Alzheimer Res. 2014;11:232–8. 24484278 10.2174/1567205011666140131120922 PMC4066812

[50] Schürmann B. Association of the Alzheimer’s Disease Clusterin Risk Allele with Plasma Clusterin Concentration. J Alzheimers Dis, 25:421–4. 21422520 10.3233/JAD-2011-110251

[51] Thambisetty M, An Y, Kinsey A, Koka D, Saleem M, Güntert A, Kraut M, Ferrucci L, Davatzikos C, Lovestone S, Resnick SM. Plasma clusterin concentration is associated with longitudinal brain atrophy in mild cognitive impairment. NeuroImage. 2012;59:212–7. 21824521 10.1016/j.neuroimage.2011.07.056 PMC3425349

[52] Schrijvers EM, Koudstaal PJ, Hofman A, Breteler MM. Plasma clusterin and the risk of Alzheimer disease. JAMA. 2011;305:1322–6. 21467285 10.1001/jama.2011.381

[53] Oh S-B, Kim MS, Park S, Son H, Kim S-Y, Kim M-S, Jo D-G, Tak E, Lee J-Y. Clusterin contributes to early stage of Alzheimer’s disease pathogenesis. Brain Pathol. 2019;29:217–31. 30295351 10.1111/bpa.12660 PMC8028273

[54] Wojtas AM, Kang SS, Olley BM, Gatherer M, Shinohara M, Lozano PA, Liu C-C, Kurti A, Baker KE, Dickson DW Loss of clusterin shifts amyloid deposition to the cerebrovasculature via disruption of perivascular drainage pathways. Proceedings of the National Academy of Sciences 2017, 114:E6962–E6971. 10.1073/pnas.1701137114 PMC5565413 28701379

[55] Demattos RB, O’Dell MA, Parsadanian M, Taylor JW, Harmony JAK, Bales KR, Paul SM, Aronow BJ, Holtzman DM. Clusterin promotes amyloid plaque formation and is critical for neuritic toxicity in a mouse model of Alzheimer’s disease. Proceedings of the National Academy of Sciences 2002, 99:10843–10848. 10.1073/pnas.162228299 PMC125060 12145324

[56] Wojtas AM, Sens JP, Kang SS, Baker KE, Berry TJ, Kurti A, Daughrity L, Jansen-West KR, Dickson DW, Petrucelli L Astrocyte-derived clusterin suppresses amyloid formation in vivo. Mol Neurodegeneration 2020, 15. 10.1186/s13024-020-00416-1 PMC7694353 33246484

[57] Chen F, Swartzlander DB, Ghosh A, Fryer JD, Wang B, Zheng H. Clusterin secreted from astrocyte promotes excitatory synaptic transmission and ameliorates Alzheimer’s disease neuropathology. Mol Neurodegeneration 2021, 16. 10.1186/s13024-021-00426-7 PMC7849119 33517893

[58] Ackley SF, Zimmerman SC, Brenowitz WD, Tchetgen Tchetgen EJ, Gold AL, Manly JJ, Mayeda ER, Filshtein TJ, Power MC, Elahi FM Effect of reductions in amyloid levels on cognitive change in randomized trials: instrumental variable meta-analysis. BMJ 2021:n156. 33632704 10.1136/bmj.n156 PMC7905687

[59] DeMattos RB, Brendza RP, Heuser JE, Kierson M, Cirrito JR, Fryer J, Sullivan PM, Fagan AM, Han X, Holtzman DM. Purification and characterization of astrocyte-secreted apolipoprotein E and J-containing lipoproteins from wild-type and human apoE transgenic mice. Neurochem Int. 2001;39:415–25. 11578777 10.1016/s0197-0186(01)00049-3

[60] Jones SE, Jomary C. Clusterin. Int J Biochem Cell Biol. 2002;34:427–31. 11906815 10.1016/s1357-2725(01)00155-8

[61] Ding X, Cao F, Cui L, Ciric B, Zhang G-X, Rostami A. IL-9 signaling affects central nervous system resident cells during inflammatory stimuli. Exp Mol Pathol. 2015;99:570–4. 26216406 10.1016/j.yexmp.2015.07.010 PMC6524950

[62] Wang C, Zhang CJ, Martin BN, Bulek K, Kang Z, Zhao J, Bian G, Carman JA, Gao J, Dongre A, IL-17 induced NOTCH1 activation in oligodendrocyte progenitor cells enhances proliferation and inflammatory gene expression. Nat Commun. 2017;8:15508. 28561022 10.1038/ncomms15508 PMC5460031

[63] Goswami R, Kaplan MH. A Brief History of IL-9. J Immunol. 2011;186:3283–8. 21368237 10.4049/jimmunol.1003049 PMC3074408

[64] Harrington EP, Bergles DE, Calabresi PA. Immune cell modulation of oligodendrocyte lineage cells. Neurosci Lett. 2020;715:134601. 31693930 10.1016/j.neulet.2019.134601 PMC6981227

[65] Kirby L, Castelo-Branco G. Crossing boundaries: Interplay between the immune system and oligodendrocyte lineage cells. Semin Cell Dev Biol. 2021;116:45–52. 33162336 10.1016/j.semcdb.2020.10.013

[66] May PC, Lampert-Etchells M, Johnson SA, Poirier J, Masters JN, Finch CE. Dynamics of gene expression for a hippocampal glycoprotein elevated in Alzheimer’s disease and in response to experimental lesions in rat. Neuron. 1990;5:831–9. 1702645 10.1016/0896-6273(90)90342-d

[67] Chi KN, Eisenhauer E, Fazli L, Jones EC, Goldenberg SL, Powers J, Tu D, Gleave ME. A phase I pharmacokinetic and pharmacodynamic study of OGX-011, a 2’-methoxyethyl antisense oligonucleotide to clusterin, in patients with localized prostate cancer. J Natl Cancer Inst. 2005;97:1287–96. 16145049 10.1093/jnci/dji252

[68] Chi KN, Higano CS, Blumenstein B, Ferrero J-M, Reeves J, Feyerabend S, Gravis G, Merseburger AS, Stenzl A, Bergman AM, Custirsen in combination with docetaxel and prednisone for patients with metastatic castration-resistant prostate cancer (SYNERGY trial): a phase 3, multicentre, open-label, randomised trial. Lancet Oncol. 2017;18:473–85. 28283282 10.1016/S1470-2045(17)30168-7

[69] Braskie MN, Jahanshad N, Stein JL, Barysheva M, McMahon KL, de Zubicaray GI, Martin NG, Wright MJ, Ringman JM, Toga AW, Thompson PM. Common Alzheimer’s disease risk variant within the CLU gene affects white matter microstructure in young adults. J Neurosci. 2011;31:6764–70. 21543606 10.1523/JNEUROSCI.5794-10.2011 PMC3176803

